# Apo-Ghrelin Receptor (apo-GHSR1a) Regulates Dopamine Signaling in the Brain

**DOI:** 10.3389/fendo.2014.00129

**Published:** 2014-08-18

**Authors:** Andras Kern, Cristina Grande, Roy G. Smith

**Affiliations:** ^1^Department of Metabolism and Aging, Scripps Research Institute Florida, Jupiter, FL, USA

**Keywords:** ghrelin, growth hormone secretagogue receptor, dopamine, heterodimers, hypothalamus

## Abstract

The orexigenic peptide hormone ghrelin is synthesized in the stomach and its receptor growth hormone secretagogue receptor (GHSR1a) is expressed mainly in the central nervous system (CNS). In this review, we confine our discussion to the physiological role of GHSR1a in the brain. Paradoxically, despite broad expression of GHSR1a in the CNS, other than trace amounts in the hypothalamus, ghrelin is undetectable in the brain. In our efforts to elucidate the function of the ligand-free ghrelin receptor (apo-GHSR1a), we identified subsets of neurons that co-express GHSR1a and dopamine receptors. In this review, we focus on interactions between apo-GHSR1a and dopamine-2 receptor (DRD2) and formation of GHSR1a:DRD2 heteromers in hypothalamic neurons that regulate appetite, and discuss implications for the treatment of Prader–Willi syndrome (PWS). GHSR1a antagonists of distinct chemical structures, a quinazolinone and a triazole, respectively, enhance and inhibit dopamine signaling through GHSR1a:DRD2 heteromers by an allosteric mechanism. This finding illustrates a potential strategy for designing the next generation of drugs for treating eating disorders as well as psychiatric disorders caused by abnormal dopamine signaling. Treatment with a GHSR1a antagonist that enhances dopamine/DRD2 activity in GHSR1a:DRD2 expressing hypothalamic neurons has the potential to inhibit the uncontrollable hyperphagia associated with PWS. DRD2 antagonists are prescribed for treating schizophrenia, but these block dopamine signaling in all DRD2 expressing neurons and are associated with adverse side effects, including enhanced appetite and excessive weight gain. A GHSR1a antagonist of structural class that allosterically blocks dopamine/DRD2 action in GHSR1a:DRD2 expressing neurons would have no effect on neurons expressing DRD2 alone; therefore, the side effects of DRD2 antagonists would potentially be reduced thereby enhancing patient compliance.

## Introduction

The orexigenic hormone ghrelin is produced in stomach and influences feeding behavior, metabolism, pulsatile growth hormone (GH) release, and immune function (1–4). Ghrelin’s action is regulated by the GH secretagogue receptor (GHSR1a, *aka* the ghrelin receptor) that was originally identified by reverse pharmacology using a small molecule, MK-0677, developed to rejuvenate the GH/insulin-like growth factor axis in elderly subjects (5, 6). GHSR1a belongs to the Class A G-protein coupled receptor (GPCR) family. In isolation, under defined conditions, GHSR1a couples to Gα_q_ resulting in activation of phospholipase C (PLC), inositol trisphosphate (IP_3_), and mobilization of [Ca^2+^]_i_ (7).

The precise physiological function of ghrelin remains to be defined. Traditionally ghrelin was believed to control appetite and facilitate excessive weight gain in response to a high-fat diet, but recent findings question these conclusions. Studies in congenic C57BL/6J *ghrelin* knockout (KO) and *ghsr* KO mice showed food intake is independent of ghrelin signaling, and that the absence of ghrelin fails to protect mice from diet-induced obesity (8–10). Indeed, recent results in transgenic mice where ghrelin producing cells were selectively ablated confirm these findings (11). Acute stimulation of food intake in ghrelin-cell ablated mice requires doses of exogenous ghrelin that produce plasma ghrelin concentrations many-fold higher than the endogenous concentrations found in wildtype mice, suggesting endogenous ghrelin is not a critical regulator of food intake. With prolonged calorie restriction ghrelin-cell ablated mice exhibit profound hypoglycemia (11). Similarly, profound hypoglycemia was reported by the same group in calorie-restricted ghrelin-deficient mice generated by ablating medium chain fatty acid acyl-transferase that is essential for converting the inactive 28-aminoacid ghrelin peptide into its biologically active form (12). Injection of ghrelin or GH rescued the hypoglycemia. Based on the results from these two transgenic mouse models, the authors concluded that ghrelin’s major metabolic role is to regulate blood glucose under conditions of famine.

GHSR1a is expressed broadly in the brain and localized mainly in the hippocampal structures, hypothalamus, midbrain, cortex, and amygdala (13). These findings led us to investigate possible interactions of GHSR1a with dopamine receptors. By employing *Ghsr-IRES-tau-GFP* mice, we showed that subsets of neurons in the midbrain and hippocampus co-express GHSR1a and dopamine receptor-1 (DRD1) (14), and in the hypothalamus subsets co-express GHSR1a and dopamine receptor-2 (DRD2) (15). *In vitro* studies illustrated the formation of GHSR1a:DRD1 heteromers, and treatment with ghrelin and dopamine in combination augmented cAMP accumulation (14). Again, using *Ghsr-IRES-tau-GFP* mice, we identified subsets of hypothalamic neurons that co-express GHSR1a and dopamine-2 receptor (DRD2) (15). DRD2 signaling influences feeding frequency and volume, and mutations in *DRD2* are associated with human obesity (16–20). In this review, we describe the co-expression of GHSR1a with dopamine receptors in neurons of the CNS, the dependence on GHSR1a for dopamine/DRD2 suppression of appetite and implications for the uncontrollable hyperphagia associated with Prader–Willi syndrome (PWS).

## Ghrelin Activation of Hypothalamic Neurons Enhances Dopamine Release from Midbrain Dopaminergic Neurons

Pharmacological doses of ghrelin activate extra-hypothalamic neurons implicating a role for ghrelin in memory, addiction, depression, and neuroprotection (21–27). Indeed, *ghsr*^−^*^/^*^−^mice exhibit impaired contextual memory (28). In addition, improvements in spatial memory induced by exogenous ghrelin are inhibited by a dopamine-1 receptor (DRD1) antagonist (29). Ghrelin also augments cocaine-induced locomotor activity and the rewarding effects of alcohol, consistent with effects on dopamine signaling (30, 31). Although ghrelin administration induces dopamine release (30), there is no evidence that ghrelin has direct access to midbrain dopaminergic neurons (32). Exogenously applied ghrelin activates c-Fos expression in the arcuate nucleus, paraventricular nucleus (PVN), and lateral hypothalamus (LH), but not in GHSR1a expressing neurons of the midbrain or hippocampus (33, 34). Tracing studies with inactivated rabies virus show that neurons in the ventral tegmental area (VTA) and pars compacta of the substantia nigra (SNpc) receive projections from the PVN and LH (33–35). Furthermore, infusion of ghrelin into the LH, but not in the VTA, stimulates orexin release, which activates orexin receptors on VTA neurons causing release of dopamine (36). Neuroanatomy, neuropharmacology, and cell biology studies indicate VTA dopaminergic neurons innervate the hippocampus (37–41). These collective observations are consistent with exogenous ghrelin inducing hippocampal plasticity indirectly by first activating neurons in the LH and PVN that in turn enhance dopamine release from the VTA resulting in activation of hippocampal neurons. However, these results obtained using pharmacological doses of ghrelin cannot simply be extrapolated to predict the physiological role of endogenous ghrelin. A recent series of studies in mice and sheep indicate that other than trace amounts in the hypothalamus, endogenous ghrelin is not present in the CNS (42–45). This is remarkable given the broad distribution of GHSR1a in the brain and underlines a potential role for GHSR1a independent of ghrelin, which led us to speculate that apo-GHSR1a regulates neuronal function through protein–protein interactions.

## GHSR1a and DRD2 are Co-Expressed in Hypothalamic Neurons Resulting in Modification of Canonical Dopamine Signaling

**Figure 1:**
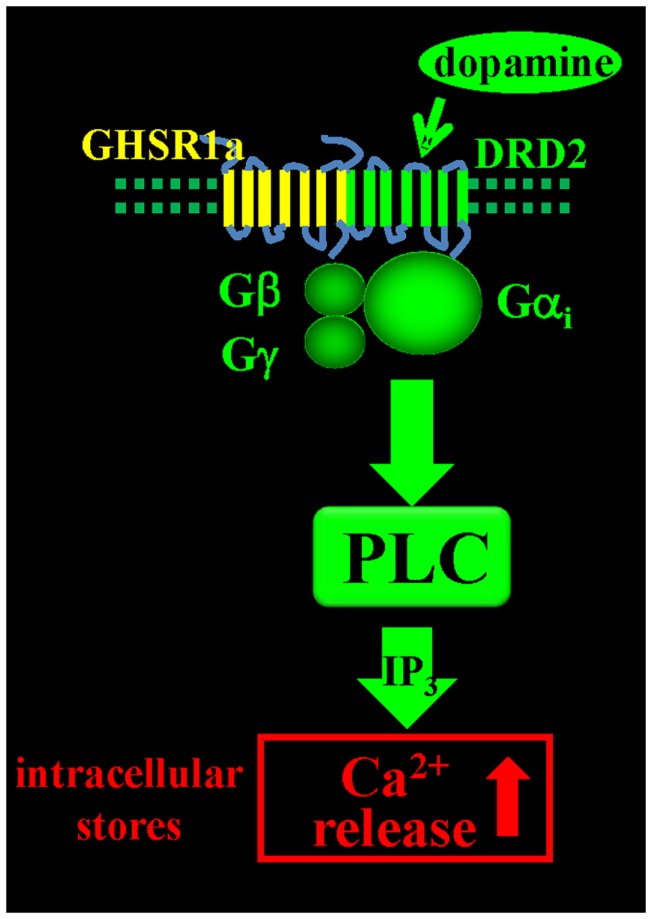
**Dopamine-induced [Ca^2+^]_i_ mobilization via GHSR1a:DRD2 heteromers**. Dopamine activation of GHSR1a:DRD2 causes release of Ca^2+^ from intracellular stores by Gβγ dependent PLC activation and mobilization of IP_3_.

Since endogenous ghrelin is undetectable in the brain, we addressed the question of what biological role un-liganded GHSR1a (apo-GHSR1a) might play by focusing on the function of hypothalamic neurons that co-express GHSR1a and DRD2. To determine the impact of expression of apo-GHSR1a on DRD2 signaling, we initially used HEK293 cells where we could control the relative expression levels of the two receptors and investigate signal transduction pathways. Dopamine activation of DRD2 in HEK293 cells results in canonical DRD2 coupling to Gα_i/o_ and suppression of intracellular cAMP accumulation without mobilization of intracellular Ca^2+^ ([Ca^2+^]_i_) (46). However, when GHSR1a is expressed with DRD2, treatment with dopamine, or the DRD2 agonist quinpirole results in [Ca^2+^]_i_ mobilization. When DRD2 is expressed with the motilin receptor, which is closely related to GHSR1a and is also coupled to Gα_q_, neither dopamine nor quinpirole treatment mobilizes [Ca^2+^]_i_, illustrating the significance of specific interactions between GHSR1a and DRD2. When GHSR1a is co-expressed with DRD2, dopamine-induced [Ca^2+^]_i_ release is blocked by: pertussis toxin, Gβγ antagonists, PLC inhibitor, thapsigargin, and IP_3_ receptor blocker. We conclude that dopamine activation causes liberation of Gβγ subunits from Gα_i/o_, activation of PLC, IP_3_ mobilization, and release of [Ca^2+^]_i_ (Figure 1). Importantly, this mechanism is activated in the complete absence of ghrelin. A mechanism involving down-stream cross-talk between GHSR1a and DRD2 caused by GHSR1a basal activity was ruled out. Blocking GHSR1a basal activity with Gα_q_ siRNA or GHSR1a point mutants that lack constitutive activity failed to inhibit dopamine-induced [Ca^2+^]_i_ release. The lack of cross-talk between the two receptors supports the hypothesis that GHSR1a and DRD2 co-expression results in non-canonical DRD2 signal transduction that is dependent on allosteric interactions between GHSR1a and DRD2 protomers. These results were recapitulated in DRD2 expressing SH-SY5Y neuroblastoma cells engineered to stably express GHSR1a, and in primary cultures of hypothalamic neurons (15), confirming that non-canonical DRD2 signaling is dependent upon interactions between GHSR1a and DRD2.

## DRD2 Agonist Suppression of Feeding Behavior is Dependent on GHSR1a and Formation of GHSR1a:DRD2 Heteromers in Hypothalamic Neurons

The change in canonical to non-canonical DRD2 signal transduction when GHSR1a is co-expressed with DRD2 implicates allosteric interactions between GHSR1a and DRD2 as a result of GHSR1a:DRD2 heteromer formation. It is well documented, based mainly on *in vitro* experiments, that GPCRs form homomers and heteromers, and that in heteromers one protomer in the complex allosterically modifies signaling of the other (47, 48). To test if modification of signal transduction is a consequence of a physical interaction between GHSR1a and DRD2, time resolved fluorescence resonance energy transfer (Tr-FRET) experiments were conducted in HEK293 cells (49, 50). The high sensitivity of Tr-FRET where SNAP- or CLIP-tags are introduced at the N-terminus of GHSR1a and DRD2 allowed monitoring of GHSR1a:GHSR1a and GHSR1a:DRD2 heteromer formation at physiological concentrations on the surface of living cells (15).

When SNAP-GHSR1a is expressed in the presence of untagged DRD2 at a 1:1 ratio the Tr-FRET signal generated by GHSR1a homomers was reduced by ~50%, consistent with formation of GHSR1a:DRD2 heteromers. As further confirmation of direct interactions and heteromer formation, Tr-FRET assays were performed using SNAP-GHSR1a and CLIP-DRD2 in a receptor titration assay. High Tr-FRET signals were detected over a wide range of receptor expression, confirming GHSR1a:DRD2 heteromerization. To test if formation of heteromers was functionally relevant, Tr-FRET results were compared to the magnitude of dopamine-induced [Ca^2+^]_i_ mobilization at various ratios of GHSR1a to DRD2. Dopamine-induced [Ca^2+^]_i_ release correlated with the concentration of GHSR1a:DRD2 heteromers. Indeed, in support of an allosteric mechanism, the GHSR1a neutral antagonist, JMV2959, inhibited dopamine-induced [Ca^2+^]_i_ mobilization without disrupting heteromer formation; similarly, a DRD2 antagonist attenuated ghrelin signaling (15). Collectively, these data show that in cell lines modification of canonical DRD2 signaling is dependent on formation of GHSR1a:DRD2 heteromers (15).

**Figure 2:**
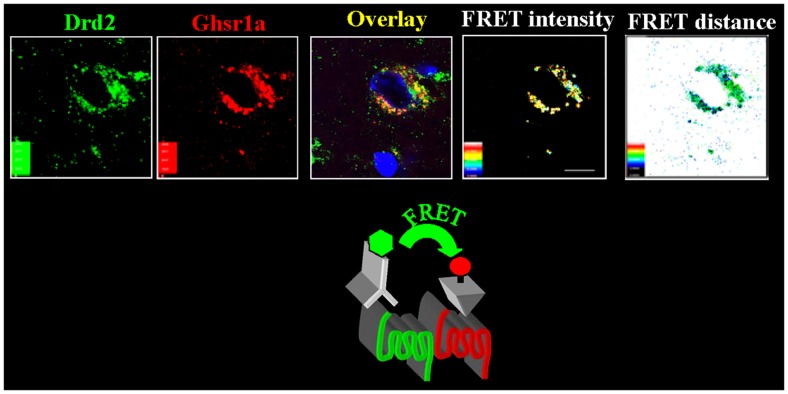
**In hypothalamic neurons GHSR1a and DRD2 form heteromers**. Hypothalamic brain slices from *Ghsr*^+^*^/^*^+^ mice were used to identify GHSR1a:DRD2 heteromers. Confocal FRET microscopy show that GHSR1a [identified using red fluorescent ghrelin (Cy5)] and DRD2 [in green, identified by immunofluorescent DRD2 monoclonal antibody (Cy3)] are in close proximity at a distance of 5–6 nm and FRET intensity 0.4–0.6 (15).

To test if GHSR1a:DRD2 heteromer formation has physiological relevance, we sought evidence for the presence of the heteromers in native brain tissue. We applied confocal FRET microscopy on mouse hypothalamic neurons using a fluorescently labeled ghrelin analog to visualize GHSR1a, and a fluorescently tagged DRD2 monoclonal antibody to localize DRD2. In hypothalamic neurons from *Ghsr*^+^*^/^*^+^ mice, confocal FRET microscopy analysis shows GHSR1a and DRD2 in close proximity within 5 nm, consistent with heteromer formation (Figure 2). In striatal neurons of *Ghsr*^+^*^/^*^+^ mice, the confocal FRET signal is very weak indicating the absence of heteromers in the striatum. In hypothalamic neurons of *Ghsr^−/−^* mice, a confocal FRET signal is not detected confirming the specificity of imaging GHSR1a:DRD2 heteromers in *Ghsr* + + mice (15).

**Figure 3:**
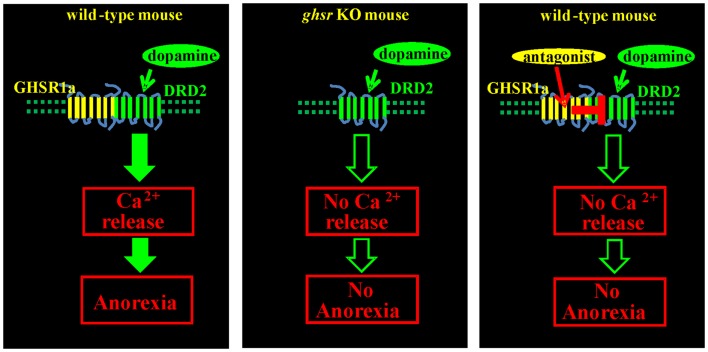
**Anorexigenic effect of a DRD2 agonist is mediated by GHSR1a:DRD2 heteromers**. Treatment of fasted *Ghsr*^+^*^/^*^+^ mice with the DRD2 agonist cabergoline inhibits food intake. *Ghsr^−/−^*mice, and *Ghsr*^+^*^/^*^+^ mice treated with the ghrelin receptor neutral antagonist, JMV2959, are refractory to the anorexigenic effects of cabergoline; hence, inhibition of food intake by cabergoline is dependent upon allosteric interactions between GHSR1a and DRD2.

The physiological importance of GHSR1a:DRD2 interactions was tested by monitoring food intake of fasted *Ghsr*^+^*^/^*^+^ and *Ghsr^−/−^*mice following treatment with the selective DRD2 agonist cabergoline. In *Ghsr*^+^*^/^*^+^ mice, food intake was markedly inhibited by cabergoline compared to vehicle treated animals, but *Ghsr^−/−^*mice were refractory to cabergoline-induced anorexia; hence, the anorexigenic activity of DRD2 is dependent upon GHSR1a. To test if endogenous ghrelin played a role, food intake was compared in *Ghrelin*^+^*^/^*^+^ and *Ghrelin^−/−^*mice treated with cabergoline. Cabergoline inhibited food intake in both genotypes, showing that the anorexigenic activity of the DRD2 agonist is dependent on GHSR1a but not on ghrelin. Having shown in cells co-expressing GHSR1a and DRD2 that the GHSR1a neutral antagonist, JMV2959, inhibits dopamine-induced [Ca^2+^]_i_ mobilization, mice were pretreated with JMV2959 before cabergoline injection. JMV2959 inhibited the anorexigenic effect of cabergoline in *Ghsr*^+^*^/^*^+^ mice, but not in *Ghsr^−/−^*mice; thus the antagonistic action of JMV2959 on DRD2 activity is dependent on GHSR1a. These data illustrate that allosteric interactions between GHSR1a and DRD2 as a consequence of heteromer formation are physiologically relevant mediators of feeding behavior (Figure 3) (15).

Our results support the concept that heteromerization of GPCRs is an important mechanism for regulating GPCR function. Many GPCRs function as oligomeric complexes, where receptors physically interact to form homo- or heteromers. These protein–protein interactions stabilize specific conformations of GPCRs to activate specific down-stream effectors and signaling pathways (51–54). For example, ghrelin inhibition of insulin release from pancreatic beta-cells is mediated by GHSR1a coupling to Gα_i_ rather than canonical coupling through Gα_q_ (55); this modification in G-protein coupling is a consequence of formation of heteromers formed between GHSR1a and somatostatin receptor-5 (56). Heterodimers formed between GHSR1a and melanocortin-3 receptor has also been reported, and a combination of *in situ* hypridization and immunohistochemistry indicated co-localization of the receptors in hypothalamic neurons (57).

Examples of dimerization of GPCRs *in vitro* are well documented, but the physiological relevance is repeatedly questioned because of the paucity of data illustrating dimerization in native tissues. However, we show by applying FRET confocal microscopy the presence of GHSR1a:DRD2 heteromers in native hypothalamic neurons and further that inhibition of food intake by a DRD2 agonist is dependent on GHSR1a. Formation of the heteromeric complex results in non-canonical signaling dependent on Gβγ activation of PLC and mobilization of [Ca^2+^]_i_. This occurs in the absence of ghrelin, showing apo-GHSR1a is capable of allosterically modifying DRD2 signaling. The allosteric interaction within the GHSR1a:DRD2 heteromer is altered by the presence of a neutral GHSR1a antagonist (15); hence, the conformation of one protomer influences the signaling properties of the other. These findings demonstrate an important role for apo-GHSR1a in the brain and resolve the paradox that GHSR1a is expressed in brain areas not accessible to peripherally produced ghrelin, and where there is no evidence of ghrelin production.

## Implications of GHSR1:DRD2 Heteromers in Obsessive Eating Associated with Prader–Willi Syndrome

Prader–Willi syndrome is a genetic disorder occurring in approximately 1 in 10,000 births and is associated with parent of origin imprinting. In normal subjects, maternal 15q11.2 is imprinted, but the paternal chromosome has a working copy of 15q11.2. Patients with PWS lack a working copy because of paternal deletion (70% of cases), maternal uniparental disomy (mUPD, 25% of cases), or imprinting errors in the imprinting center (IC, 5% of cases) (58). The deletion or lack of expression of 15q11.2 on the paternal chromosome results in a complex multi-systemic disorder. The phenotype includes short stature, low muscle tone, uncontrollable appetite, incomplete sexual development, and impaired cognition. The behavioral characteristics fall into the spectrum of autism spectrum disorders. In narrowing down the DNA region responsible for PWS, it was found that individuals with paternal deletion of the *SNORD116* gene cluster had all the major characteristic features of PWS (59). This locus encompasses a long non-coding RNA transcript that is processed into multiple small nuclear RNAs, as well as spliced exons of host gene *116G* (60). The expression of *Snord116* in mice becomes progressively more prominent in the arcuate nucleus, PVN, and ventromedial nucleus of the hypothalamus as the mice mature (61); these particular areas express GHSR1a and are involved in regulation of appetite mediated by DRD2.

While GH replacement is frequently used to treat impaired growth and hypotonia associated with PWS, the most distinguishing feature, for which there is no medical therapy, is the involuntary and uncontrollable chronic feeling of hunger, combined with a slow metabolism that leads to excessive eating and life-threatening obesity (62–64). The major theory proposed for explaining the insatiable appetite and excessive weight gain associated with PWS is elevated circulating levels of the orexigenic hormone ghrelin in the blood (65–67). Despite this, the direct approach of treating PWS patients with a GHSR1a antagonist, to our knowledge, has not been tested; this is likely because early clinical studies where GHSR1a antagonists were tested as antiobesity agents in normal subjects proved disappointing. The age-dependent (7 months to 5 years) transition to hyperphagia in PWS does not correlate with a change in ghrelin levels (68). However, as PWS children progress into adulthood hyperphagia becomes more severe; notably, the postprandial decrease in ghrelin observed in normal subjects is absent (69). Indeed, this is a most important distinction between normal and PWS adults. The postprandial nadir in endogenous ghrelin observed in normal subjects provides a mechanism for GHSR1a re-sensitization, and a satiety signal to tell the brain – stop eating! Indeed, the overall phenotype of PWS can be interpreted as impaired GHSR1a re-sensitization and as consequence low levels of active GHSR1a.

Prader–Willi syndrome patients present with high ghrelin and low GH levels; however, the anterior pituitary gland appears structurally normal and produces GH, suggesting impaired hypothalamic-pituitary signaling. Since GHSR1a agonists, including ghrelin, stimulate GH release, a possible explanation, other than GHSR1a desensitization, is that *GHSR* expression is attenuated. Expression of *GHSR* is positively regulated by thyroid hormone and estradiol, and negatively by cortisol (70). Indeed, hypothyroidism, hypogonadism, and elevated cortisol are all associated with PWS (71). Although not assessed quantitatively, GHSR1a expression appears normal in brains from deceased PWS patients (72). Nevertheless, compared to normal subjects, PWS patients elicit attenuated GH release in response to a GHSR1a agonist (73). High plasma concentrations of ghrelin should result in hyperglycemia and insulin resistance, but these metabolic changes are not consistently observed in PWS (73, 74). Lowering ghrelin levels by administering somatostatin or octreotide had no effect on appetite, weight gain, or behavior in PWS subjects (75–77). However, this does not preclude a role for ghrelin, because these compounds also suppress secretion of gastrointestinal anorexigenic hormones (76).

A recent report on the *Snord116*^±^ mouse model of PWS showed that different inhibitors of GHSR1a failed to inhibit food intake (78) and that the involvement of some new pathway is linked to changes in feeding and psychiatric behavior (79). The authors concluded that ghrelin signaling is not involved in PWS and perhaps the elevated plasma ghrelin concentration is playing a compensatory role in PWS subjects. These findings support our suggestion that elevated endogenous ghrelin results in GHSR1a desensitization and lower levels of active GHSR1a. Low GHSR1a levels on the plasma membrane of hypothalamic neurons would predictably cause hyperphagia because as described above the appetite suppressing effect of DRD2 agonists is dependent on molecular interactions with apo-GHSR1a and DRD2 (15). Indeed, *ghsr*^−/−^ mice are completely resistant to the anorexigenic effect of the DRD2 agonist cabergoline. Down-stream, cabergoline increases expression and signaling of brain-derived neurotrophic factor (BDNF). BDNF is one of the most important suppressors of food intake, and BDNF is low in PWS (80, 81).

Endogenous dopamine signaling via DRD2 inhibits excessive food intake and is dependent upon apo-GHSR1a and formation of GHSR1a:DRD2 heteromers. Formation of heteromers results in non-canonical DRD2 signal transduction and suppression of food intake. We suggest that persistently elevated circulating ghrelin in PWS reduces accumulation of GHSR1a on the plasma membrane of hypothalamic neurons. Low concentrations of GHSR1a contribute to hyperphagia by lowering the concentration of GHSR1a:DRD2, thereby attenuating satiety signals regulated by dopamine. The feasibility of targeting heterodimers formed between GHSR1a and DRD2 to modify non-canonical dopamine signaling was illustrated by us previously (15). This finding provides an approach to suppress or augment dopamine signaling selectively in neurons that express GHSR1a:DRD2 through an allosteric mechanism. Indeed, we have identified GHSR1a antagonists belonging to distinct structural classes, quinazolinones and triazoles that respectively enhance and inhibit DRD2 signaling through GHSR1a:DRD2 heteromers. Since these heteromers are found in a hypothalamic regions involved in development of PWS, GHSR1a antagonists that enhance DRD2 signaling are potential candidates for therapeutic intervention to suppress appetite. Targeting dopamine signaling in GHSR1a:DRD2 expressing neurons with GHSR1a antagonists provides the opportunity to design drugs that selectively target neurons co-expressing GHSR1a and DRD2 without affecting neurons expressing DRD2 alone.

## Summary

In subsets of hypothalamic neurons that co-express GHSR1a and DRD2, GHSR1a:DRD2 heteromers are formed. Formation of these heteromers results in allosteric modification of the conformation of DRD2, thereby causing non-canonical signal transduction in response to dopamine. In contrast to canonical DRD2 signal transduction that involves suppression of cAMP accumulation, the non-canonical pathway results in Gβγ_γ_ subunit-dependent activation of PLC and mobilization of [Ca^2+^]_i_. The physiological relevance of GHSR1a:DRD2 heteromers is illustrated by experiments in *Ghsr*^+/+^, *Ghsr*^−^*^/^*^−^, and *Ghrelin*^−^*^/^*^−^ mice. DRD2 agonism produces anorexia in *Ghsr*^+^*^/^*^+^ and *Ghrelin*^−^*^/^*^−^ mice, but not in *Ghsr*^−^*^/^*^−^mice. Hence, the anorexigenic effects of DRD2 agonists are dependent on GHSR1a, but not ghrelin. Further, *Ghsr*^+^*^/^*^+^ mice treated with a neutral GHSR1a antagonist (the triazole, JMV2959) are resistant to DRD2 agonist-induced anorexia. Hence, pharmacological intervention with a GHSR1a antagonist according to structure, triazole vs. quinazolinone will respectively allosterically block or enhance dopamine signaling in neurons expressing GHSR1a:DRD2 heteromers without affecting signaling in neurons expressing DRD2 alone. These results show the potential of developing drugs that selectively act on subsets of neurons that express GHSR1a:DRD2 heteromers for treating obsessive eating disorders such as in PWS and for psychiatric disorders associated with irregularities in dopamine signaling.

## Conflict of Interest Statement

The authors declare that the research was conducted in the absence of any commercial or financial relationships that could be construed as a potential conflict of interest.
